# A Manual of Diseases of the Throat and Nose

**Published:** 1881-03

**Authors:** 


A Manual of Diseases of the Throat and Nose. By Francke Huntington Bosworth, A.M., M.D., Lecturer on Diseases of the Throat in the Bellevue Hospital Medical College, and Physician-in-Charge of Clinic for Diseases of the Throat in the Out door Department of Bellevue Hospital, etc. Wm. Wood & Co., New York. 1881.

This is a valuable book on the subject upon which it treats, and the specialist on diseases of the throat and nose cannot call his library complete without it. It is gotten up in Messrs. Wm. Wood & Co.'s usual happy style, and no doubt will meet with ready sale.

